# Association between dietary inflammatory index and bone density in lactating women at 6 months postpartum: a longitudinal study

**DOI:** 10.1186/s12889-019-7409-6

**Published:** 2019-08-09

**Authors:** Yalin Zhou, Xiaoyu Zhu, Minjia Zhang, Yong Li, Wei Liu, Hanming Huang, Yajun Xu

**Affiliations:** 1 Department of Nutrition and Food Hygiene, School of Public Health, Peking University, NO.38 Xueyuan Road, Beijing, 100083 China; 2 Beijing Northern Hospital, NO.10 Chedaogou Road, Beijing, China; 3 Beijing Key Laboratory of Toxicological Research and Risk Assessment for Food Safety, Peking University, Beijing, NO.38 Xueyuan Road, Beijing, 100083 China

**Keywords:** Dietary inflammatory index, Bone density, Chinese lactating women, Prospective study

## Abstract

**Background:**

Chronic inflammation contributes to the risk of osteoporosis and fracture. Dietary Inflammatory Index (DII), a novel method appraising the inflammatory potential of diet, has been utilized to examine the association between diet and bone health among postmenopausal women or the elderly. However, its relationship with bone density (BD) in lactating women has not been studied.

**Methods:**

The prospective study was conducted to assess the possible association between DII and maternal BD during lactation. We enrolled 150 lactating women in the cohort. Participants were measured ultrasonic BD as baseline values at 1 month postpartum. After five-month follow up, the participants’ BD were measured again. DII scores were calculated from semi-quantitative food frequency questionnaires (FFQ) and divided into tertiles. We compared the differences in the changes of BD at 6 months postpartum without or with adjustment for potential covariates across the tertiles.

**Results:**

The women in Q1 of DII scores had less bone mass loss than those in Q2 and Q3 without adjustment for any covariates (*p* < 0.01); after adjusting demographic characteristics such as BMI (kg/m^2^) at 6 months postpartum, educational level, metabolic equivalent (MET), daily energy intake (kcal/d), we found that participants in the highest tertile of DII scores had much more bone loss than those in the lowest tertile (*p* = 0.038). However, in the test for trend, no significant association between DII and the changes of maternal BD at 6 months postpartum was observed.

**Conclusions:**

Chinese lactating women with higher DII scores have more bone mass loss; however significant differences and trends are attenuated and/or disappear depending on covariates and confounders that are taken into account in statistical analysis. The further study should be conducted in larger population to explore whether the significant association between DII and BD exists in Chinese lactating women.

## Background

On average, Chinese women reach the peak bone mass (PBM) at the age of 30 [1] and that age is also the peak period of their child-bearing [2]. During lactation, substantial calcium enters into human milk for supporting infants bone development [3]. In order to maintain the calcium balance in milk, calcium from maternal bones is dissolved, which leads to maternal PBM loss. If calcium is not supplied adequately, the bone loss can be further exacerbated. However, regrettably, according to the data on Chinese National Nutrition and Health Surveillance, more than 50% of breastfeeding women consumed less milk than the recommendation, which could not meet the demands of calcium of the population. In addition, the data also showed that Chinese breastfeeding women had lower intake of vitamin D and calcium. It is no doubt that the increased loss and decreased intake of calcium would significantly increase the lack of PBM [4]. Therefore, Chinese lactating women are at high risk of the lack of PBM reserve.

It is well documented that inflammation is associated with the disturbance of bone metabolism. Chronic inflammation also contributes to the risk of osteoporosis and fracture [5–7]. As we all know, estrogen plays a vital role in alleviating inflammation. During lactation, prolactin suppresses the release of estrogens in lactating women [8, 9]. For postmenopausal women, the sharp decrease in estrogen is the leading risk factor for postmenopausal osteoporosis [10]. Therefore, it is hypothesized that inflammation may be a potential pathway through which reduced level of estrogen detriments bone health [11, 12].

Diet can regulate the inflammatory level through pro- or anti- inflammatory mechanisms [13–15]. It has been reported that diet characterized by high intake of red meats, high-fat milk, refined grains, processed meats, and sweets as well as candies and soft drinks potentially increased the inflammatory level [16]. In comparison, healthy diet with high intake of fruits, vegetables, whole grains negatively relates to inflammatory factors (CRP, IL-6) [17]. In addition, some well-established dietary patterns (DPs) have been reported to exert impacts on levels of inflammatory markers [18]. Mediterranean Diet [19], Health Index Diet [20, 21], and DASH [22, 23] have anti-inflammatory potential. A meta-analysis of the effect of healthy DPs on inflammatory biomarkers showed that the consumption of a healthy DP (plant food based with mostly fruit, vegetables, and wholegrains and little red meat) was linked to significant reductions in CRP (− 0.75, 95%CI: − 1.16, − 0.35) [20]. In another meta-analysis, the findings showed that the diet with low fat intake could cause reduced levels of CRP in patients with metabolic syndrome (− 0.98, 95%CI: − 1.60, − 0.35), [24]. Another prospective study on the long-term associations between DPs and CRP status also demonstrated that a DP with higher intake of vegetables and vegetable oils could reduce the risk of elevated CRP [25].

Recently, there has been increasing studies on evaluating inflammatory effects of diet by using DII [14, 26]. DII is a scoring algorithm based on the review of extensive literature that assessed the relationship between 6 common inflammatory markers (IL-1β, IL-6, TNF-α, CRP, IL-4, IL-10) and foods or nutrients [16].

The strong association between DII and well-known inflammatory markers has been validated by a majority of studies [16, 17, 26–29]. DII provides a novel prospect to appraise the effects of diets on pathogenesis and development of diseases, especially chronic metabolic diseases. In a recent case-control study, the results suggested that DII scores were positively associated with the risk of fracture among Chinese postmenopausal women, that is, the postmenopausal women with higher DII scores were prone to higher risk of fractures [16]. Similarly, in another prospective study, high intake of pro-inflammatory diets increased the risk of fracture among US adults [30].

Given the high risk of PBM deficiency and the lack of study on the correlation between DII and BD among Chinese lactating women, we conducted the current study with the aim to use DII assessing the diet quality of lactating women and then research the relationship between DII and maternal BD during lactation in China.

## Methods

### Participants

The cohort was set up between November 1, 2016 and November 1, 2017 in Beijing, China. Participants who took part in postpartum exam at 1 month postpartum in Beijing Northern Hospital were recruited. When women came to the designed hospital for postpartum exam on a regular basis at 1 month postpartum, our trained researchers would tell them some information about our study and give informed consent. Of these, 200 women at 1 month postpartum agreed to participate in baseline study. Inclusion criteria for lactating women were as followings: being at 1 month postpartum, being apparently healthy with no acute or chronic diseases. Women who were illiterate and had difficulty with oral communication were excluded. 31 women were excluded because of diseases seriously affecting the bone metabolism, such as history of thyroid, renal failure, malignancy, rheumatoid arthritis, hormone replacement therapies. We enrolled 154 women in the longitudinal analysis after exclusion of 15 women who lost follow-up.

### The sample size

We calculated the sample size by using the formula:

$$ n=\frac{{\left({z}_a\sqrt{2\overline{p}\overline{q}+{z}_{\beta }}\sqrt{p_0{q}_0+{p}_1{q}_1}\right)}^2}{{\left({p}_1-{p}_0\right)}^2} $$

According to data on calcaneal ultrasound BD of 661,864 adults in Beijing in 2015, there were 32.4% of bearing-child women suffered from bone mass loss [31]. We hypothesized that high DII increased the risk of bone loss. Generally, when the relative risk (RR) is 1.5–2.9, it is believed that there might be a moderate correlation between disease and exposure. In the current study, we set RR = 2.0, *α* = 0.05 and *β* = 0.10.

$$ {\displaystyle \begin{array}{c}{Z}_{\alpha }=1.96,{Z}_{\beta }=1.282,{p}_0=0.324,{q}_0=1-0.324=0.676\\ {}{p}_1={p}_0\ast RR=0.648,{q}_1=1-{p}_1=0.352\\ {}\overline{p}=1/2\ast \left({p}_0+{p}_1\right)=0.486,\overline{q}=1-\overline{p}=0.514\end{array}} $$

We put the above data into the formula and concluded the sample size of 96. The final sample size should be 106–115 people considering 10–20% lost follow-up. A total of 154 women were recruited in our study, which met the demands of sample size.

### Study design

At 1 month postpartum, participants were measured ultrasonic BD as baseline values and instructed to complete a self-designed structured questionnaire including a semi-quantitative Food Frequency Questionnaire (FFQ). At 6 months postpartum, we conducted one follow-up visit after 5 months. The participants were telephoned and informed of a second measure of BD; at the same time, their information on demographic characteristics, life styles, child-bearing history, feeding styles and medical history as well as physical activity were collected using a self-designed structured questionnaire without FFQ under interviewers’ instruction. We chose 1 month postpartum as baseline due to the following reasons: firstly, the designed hospital of the study was not the hospital where the pregnant women registered and gave birth, so we could not obtain the pre-pregnant and pregnant information; secondly, women at 1 month postpartum would take routine physical examination in the designed hospital, which provided opportunities and convenience for our research; finally, in China, “sitting month” is a Chinese tradition custom for women at 1 month postpartum and the diet was special during the period, which might influence maternal bone health.

### Measurements of BD

Quantitative ultrasonometry (QUS) was used for the measure of calcaneal BD. At 1 and 6 months postpartum, the participants’ calcaneal QUS measure was carried out using the CM-200 device (Furuno Electric Nishinomiya City, Japan). This equipment included two transducers the distance between which can be regulated according to the size of subjects’ feet. One of the transducers represented the transmitter while the other one worked as the receiver. The measurement of BD was operated by a trained technician. Subjects sat on a bench with the calf perpendicular to the thigh and immersed their right heels between the two transducers. The sound waves were transmitted and then the ultrasound variable, speed of sound (SOS, m/s) was recorded and stored in the matched computer connected to the device. The final results were displayed as the T-score which was calculated by subtracting the PBM of adults of the same sex from the detected subjects’ BD and then dividing the former. Each subject was repeatedly measured for three times and the average T-score was adopted as the outcome variable. The coefficient of variation (CV) of the machine was 0.19%. The machine was calibrated by using the matched module prior to each measurement.

### Changes of maternal BD at 6 months postpartum

Changes of maternal BD were calculated via subtracting baseline T-score at 1 month postpartum from follow-up T-score at 6 months postpartum.

### Dietary assessment

Maternal dietary intake was assessed with a 48-item food semi-quantitative FFQ based on a previously validated FFQ [32] and minimally modified for being more applicable to lactating women and improving the compliance of the subjects in answering some sections. Women were administered to report their food intake frequency (how many times of dietary intake per day, per week or per month) and quantity. Food models were prepared in order to help women recall food consumption. The amounts of each food item were calculated by multiplying the frequency by the amount per time and then were transformed into daily amount. Daily mean nutrients and energy intake were calculated using the Chinese Food Composition Table, 2009 [16] and US Department of Agriculture (USDA) database for flavonoids and proanthocyandins, as well as vitamin D, and Hong Kong database for isoflavones. The women whose daily energy intake was less than 1% or more than 99% were excluded from the analysis. Four women were excluded due to the criterion. Therefore, a total of 150 women were recruited in our study.

### Dietary inflammatory index

The construction and validation of DII has been described thoroughly in studies previously published [26]. The components of DII consist of 45 food parameters including: foods, nutrients, and other bioactive components. DII was identified on the basis of food parameters’ effects on six typical inflammatory markers: CRP, IL-1β, IL-4, IL-6, IL-10, and TNF-α [26].

A standardized mean for each food parameter from representative word database [26] was subtracted from actual intake of every food parameters, and then divided by its standard deviation so as to get Z scores. In order to minimize effects of outliners or non-symmetrical distribution, Z scores subsequently were converted to proportions, then doubling and subtracting 1 [15]. The final values were multiplied by the responding inflammatory effect index and summed across all food parameters, so as to generate the overall DII. The higher DII scores meant the more pro-inflammatory effects of diets, whereas more negative values implied more anti-inflammatory effects of diet. In the current study, 27 of 45 food parameters were available for calculation of DII scores: total intake of energy, protein, carbohydrate, total fat, cholesterol, saturated fatty acids, iron for pro-inflammatory factors; there were the anti-inflammatory factors: monounsaturated fatty acids, polyunsaturated fatty acids (PUFAs), vitamin A, vitamin C, vitamin D, vitamin E, thiamine, riboflavin, niacin, fiber, β-carotene, magnesium, zinc, selenium, alcohol, flavan3-ol, flavonols, flavones, isoflavones, and anthocyanidins.

### Covariates

Covariates included body weight or height [18], body mass index [BMI, in weight (kg)/height^2^ (m)], physical activity, smoking [33], and alcohol as well as estrogen use and the use of calcium or Vitamin D or multivitamin supplements [34–36]. BMI was calculated from medical records of height and weight at 6 months postpartum. Educational status was classified in three levels: high school or below, college, postgraduate or up.

Data on infant feeding modes (the infant feeding modes during 0–6 month) were collected at 6 months postpartum. The types of infant feeding were classified as follows: 1) breastfeeding (breast milk was predominantly or exclusively given with or without a very small amount of water or/an juice); 2) mixed feeding (a mixture of breast milk and formula milk was given with or without some water or/an juice); 3) artificial feeding (formula milk was predominantly given with or without some water or/an juice) [37].

Smoking status and alcohol consumption were assessed as binary variable (yes or no). Physical activity levels were represented as METs*hour evaluated by calculating the ratio of working metabolic rate to resting metabolic rate (referred to as “METs index”) based on the 7-day total activity recall of the participants [38].

Intake of calcium and Vitamin D supplements recorded on the supplement section of the FFQ was calculated by multiplying times by amount of intake per time and evaluated as continuous variables. Multivitamin supplements were coded as yes-no variables.

### Statistical analysis

Analyses were conducted by using SPSS software version 22.0 (SPSS Inc., Chicago, IL, USA). The level of significance was *p* values<0.05. DII scores were divided in to tertiles. Sample means (SD) for continuous variables, and frequencies (percentages) for categorized variables were calculated. We compared the difference in participants’ characteristics across tertiles of DII using One-way analysis of variance (ANOVA) for continuous variables, and chi-square test was used for categorized variables. A test for linear trend was conducted by including the median value of each DII tertile as a continuous variable in the multiple liner regression models. For multiple linear analyses, three models were established for adjusting covariates: Model 1: without adjustment; Model 2: multivariate analysis was adjusted for demographic characteristics: age, BMI (kg/m^2^) at 6 months postpartum (BMI6), baseline T-score, METs, educational levels; Model 3: further adjusted for variables related to child-bearing history: parity, the mode of infant feeding, time of complementary foods. Nutritional parameters (such as calcium, Vitamin D, multivitamin supplements) were not included in the fully adjusted model, since these food parameters were already included in the calculation of DII scores. Multi-collinearity among covariates was estimated via variance inflation factor (VIF) 2 as the cutoff. No variables met the criterion and thus none was removed from models. In the end, we compared the adjusted mean (SD) changes of BD across each tertile of DII and calculated *p* for liner trend test.

None of women participated in our study had a history of smoking, drinking, or estrogen use. Therefore, we did not enter these variables into the regression analysis.

## Results

### Participants

The mean of DII scores was − 0.039 (SD = 2.16, range − 4.33 to 4.24). Demographic characteristics by DII tertiles were illustrated in Table 1. Those in the highest DII tertile (consuming the most pro-inflammatory diet) had lower daily energy intake (*p* < 0.01). Participants with higher DII scores had less parity than other participants (*p* = 0.03).

**Table 1:** Samples demographic characteristics across tertile (Q) of DII for 150 women in Beijing Northern Hospital

Characteristics	Tertiles of DII			*p* ^a^
	Q1(*n* = 49)	Q2(*n* = 51)	Q3(*n* = 50)	
Age, years	32.94 (4.67)	31.27 (3.6)	31 (4.3)	0.120
BMI6,kg/m^2^	22.2 (3.75)	22.93 (3.53)	23.5 (3.63)	0.331
Educational level, n,%				0.641
High school or below	4 (7.1)	5 (9.8)	1 (2.0)	
College	22 (45.2)	29 (56.1)	25 (49.2)	
Postgraduate or up	23 (47.6)	17 (34.1)	24 (48.8)	
Time spent for physical activity, n,%				0.379
< 1 h/week	16 (33.3)	10 (20.5)	16 (31.6)	
1-2 h/week	25 (50.0)	31 (61.4)	24 (47.4)	
2-4 h/week	6 (11.9)	6 (11.4)	10 (21.1)	
4-6 h/week	2 (4.8)	4 (6.8)	0 (0)	
T0	−1.2 (0.79)	−1.03 (0.77)	−0.8 (0.91)	0.081
T6	−1.26 (0.84)	− 1.04 (0.79)	− 0.76 (1.11)	0.127
Energy intake kcal/d	3164.37 (1009.32)	2060.79 (806.64)	1345.41 (359.32)	< 0.001
Calcium supplement, g/d	2.82 (11.42)	0.37 (0.41)	0.25 (0.28)	0.208
Vitamin D,IU/d	110.92 (144.09)	200.28 (305.16)	116.87 (172.46)	0.174
Multivitamin use, n,%				0.890
Yes	29 (58.5)	32 (62.5)	32 (63.4)	
No	20 (41.5)	19 (37.5)	18 (36.6)	
METs	10.7 (20.47)	8.8 (14.38)	3.74 (4.5)	0.142
Menarche, age, years	13.42 (1.63)	12.91 (1.79)	13.36 (1.52)	0.390
Pregnancy	1.97 (1.21)	1.58 (0.83)	1.52 (0.76)	0.100
Parity, times	1.69 (1.19)	1.27 (0.45)	1.24 (0.44)	0.030
Calf Cramp, n,%				0.181
Yes	18 (34.1)	9 (17.1)	13 (26.8)	
No	31 (65.9)	42 (73.2)	37 (65.9)	
Feeding styles (0-6 month), n,%				0.771
Breastfeeding	26 (61.9)	27 (65.9)	28 (68.3)	
Mixed feeding	14 (33.3)	11 (26.8)	9 (22.0)	
Artificial feeding	2 (4.8)	3 (7.3)	4 (9.8)	
Complementary foods, n,%				0.920
Yes	43 (88.3)	45 (87.8)	43 (85.4)	
No	6 (11.9)	6 (12.2)	7 (14.6)	
Time of Complementary food	5.56 (0.52)	5.42 (0.55)	5.55 (0.63)	0.571

Data are mean (SD) for normally distributed variables and % (n) for categorical variables.

*Note.* BMI6 = BMI (kg/m^2^) at 6 months postpartum; T0 = baseline T-score at 1 month postpartum; T6 = follow-up T-score at 6 months postpartum

^a^ One-way analysis of variance (ANOVA) was used for difference in participants’ characteristics across tertiles of DII and chi-square test for categorical variables

### General characteristics across tertiles of DII

Distribution of food groups across tertiles of DII is shown in Table 2: decreasing trends were observed for energy daily intake (*p* < 0.01), soy and soy products (*p* = 0.01), dark leafy vegetables (*p* = 0.002), light vegetables (*p* = 0.001), dark fruits (*p* = 0.003), and light fruits (*p* = 0.023).

**Table 2:** Distribution of food groups across tertiles of DII for 150 women in Beijing Northern Hospital

Food Groups	Tertiles of DII			*p* ^b^ for trend
	Q1	Q2	Q3	
Dietary energy (kcal/d)	3164.37 (1009.32)	2060.79 (806.64)	1345.41 (359.32)	0.000
Refined Cereals	442.86 (207.73)	387.22 (230.79)	341.63 (156.75)	0.587
Whole Cereals(g/d)	176.65 (153.68)	72.63 (52.80)	72.9 (130.05)	0.153
Soy and soy product(g/d)	146.09 (145.11)	81.8 (75.51)	36.19 (47.54)	0.010
Dark leafy vegetables(g/d)	894.49 (621.10)	453.71 (228.40)	293.03 (234.09)	0.002
Light vegetables(g/d)	388.55 (305.45)	163.51 (119.32)	100.23 (84.61)	0.001
Dark fruits(g/d)	232.98 (159.01)	140.37 (119.66)	46.89 (67.93)	0.004
Light fruits(g/d)	197.17 (135.86)	161.53 (105.46)	92.73 (75.12)	0.023
Poultry(g/d)	71.55 (87.22)	42.35 (84.42)	30.9 (46.67)	0.590
Meat and Processed meats(g/d)	132.56 (102.78)	93.6 (109.69)	79.18 (60.52)	0.531
Fish and aquatic products(g/d)	69.18 (37.64)	75.27 (46.99)	59.34 (41.76)	0.512
Eggs(g/d)	69.18 (37.64)	75.27 (46.99)	59.34 (41.76)	0.894
Milk and Dairy products(g/d)	281.89 (281.07)	239.59 (262.31)	144.12 (136.57)	0.744
Fungus and Alga(g/d)	38.91 (83.15)	18.34 (22.49)	9.69 (10.11)	0.542
Nuts(g/d)	24.35 (33.05)	10.83 (15.28)	9.28 (11.20)	0.384
Backed Bread(g/d)	41.75 (41.02)	45.06 (102.61)	14.84 (24.40)	0.632
Candies(g/d)	5.67 (16.16)	4.73 (11.10)	1.67 (02.53)	0.678
Junk food(g/d)	18.78 (36.87)	14.55 (32.99)	6.69 (14.90)	0.489
Fruit and vegetable juice (ml/d)	47.24 (148.24)	34.76 (66.35)	7.01 (21.63)	0.882
Soft drinks (ml/d)	79.81 (129.28)	38.23 (67.61)	29.65 (69.40)	0.723

Data are mean (SD) for normally distributed variables.

^b^ A test for linear trend was conducted by including the median value of each DII tertile as a continuous term in the simple liner regression analysis

### Relationship between changes of maternal BD at 6 months postpartum and DII

The multivariate adjusted means of changes of maternal BD at 6 months postpartum across tertiles of DII are shown in Table 3. Women in the Q1 of DII had less bone mass loss than those in Q2 and Q3 without adjustment (*p* < 0.01); after adjusting demographic characteristics such as BMI6, educational level, METs, daily energy intake (kcal/d), we found that participants in the highest tertile had much more bone loss than those in the Q1 (*p* = 0.038). To test for trend, we used DII median of each tertile as continuous variables for multivariate models; however, there was no significant liner relationship between the changes of maternal BD at 6 months postpartum and DII.

**Table 3:** Adjusted means of maternal BD changes across tertiles of DII for 150 women in Beijing Northern Hospital

Tertiles of DII scores				*p* ^c^ for trend
	Q1	Q2	Q3	
Model1^d^	0.03 (0.8)	0.01 (0.9) **	−0.09 (0.9) **	0.748
Model2^e^	0.03 (0.2)	−0.01 (0.3)	− 0.07 (0.3) *	0.669
Model3^f^	0.05 (0.3)	0.00 (0.3)	−0.1 (0.4)	0.581

^c^ A test for linear trend was conducted by including the median value of each DII tertile as a continuous term in the regression analysis

^d^Model1: Without adjustment; ^e^Model2: Multivariate models were adjusted for demographic characteristics: BMI6 (kg/m^2^), educational level, METs, daily energy intake (kcal/d), baseline T-score (T0); ^f^ Model3: Further adjusted for feeding modes, time of complementary foods

* vs Q1, *p* < 0.05, ** vs Q1, *p* < 0.01

## Discussion

To the best of our knowledge, this was the first study to focus on Chinese lactating women and evaluate the association between their BD and DII. In this cohort study, participants were measured ultrasonic BD at 1 and 6 months postpartum. We calculated their DII scores according to dietary data collected via FFQ, and explored the association between maternal DII and the changes of BMD at 6 months postpartum. The findings showed that higher DII scores were related to greater loss of BD, although the test for trend did not reach statistical significance due to some limitations.

There existed some studies involved in the correlation between DII and BMD or fracture. A longitudinal study enrolling 160,191 postmenopausal women in Women Health Institute showed that the higher DII scores (more intake of pro-inflammatory diet) increased the risk of hip fracture (HR = 1.48, 95% CI: 1.9–2.01) among white elderly women under 63 [5]. Similarly, another study on association between DII and the changes of BD revealed that elderly women with the highest DII scores had more bone mass loss (*p* = 0.01) [5]. In agreement with above-mentioned findings, a 1:1 matched case-control study conducted among 1050 Chinese elderly people (52-83y) with fractures displayed the significant correlation between DII and risk of fracture (females: OR = 2.08, 95% CI: 1.38–3.12, males: OR = 4.30, 95% CI: 1.89–9.80) [16]. The cross-sectional study conducted in 160 postmenopausal Iranian women aged 50–85 years also showed that those with higher intake of pro-inflammatory diets were subjected to lower lumbar BMD (OR = 1.64, 95% CI: 1.11–2.43) [15]. In spite of variance in participants and study design, the similar conclusion was drawn that high DII scores (higher intake of pro-inflammatory foods) has an adverse impact on bone health. Inspired by these findings, we speculated that women consuming more pro-inflammatory diets would suffer greater bone loss during lactation. In our study, we observed that lactating women with the highest DII suffered more bone loss.

Chronic inflammation is involved in the pathogenesis of cardiovascular diseases, diabetes, and cancers. It also acts as an important role in bone-related diseases, such as osteoporosis and fracture [39]. In vitro, pro-inflammatory factors (IL-6, CRP) inhibit osteoblast function and promote the proliferation, differentiation, and activation of osteoclast, which causes bone erosion and subsequently the bone mass loss [11, 40, 41]. Apart from regulating function of osteoblast and osteoclast, inflammation in intestines upregulates the synthesis of 1,25(OH)_2_D, suppresses the expression of vitamin D receptor, and hinders the absorption of calcium and phosphorus [42, 43]. Epidemiology studies demonstrated that elevated levels of inflammatory markers in serums were negatively associated with BMD [44, 45]. RCTs also verified that reduced inflammatory levels were conducive to improve bone mineral density (BMD) [46].

The relationship between DPs and bone health is widely controversial. However, the DPs typical of high intake of vegetables, fruits, whole grains, soybeans have been commonly recognized as positive factors enhancing bone health [47–50]. We hypothesized that anti-inflammatory effect of the DPs plays a crucial role. Interestingly, in the present study, we found the significantly decreasing trends for dark leafy vegetables (*p* = 0.002), light vegetables (*p* = 0.001), dark fruits (*p* = 0.003), light fruits (*p* = 0.023) across tertiles of DII. Women with lower DII scores tend to consume more fruits and vegetables, whole grains, soybean and products. In animals’ models, diet rich in phytochemicals reduced inflammatory markers in circulation, and improved anti-oxidative ability [15]. The epidemiological studies also found that Mediterranean Diet [20], Health Index Diet [20, 21], and DASH diet [51], all of which emphasized high consumption of vegetables, fruits, whole grains and low fat foods, contributed to the declined inflammatory indexes in the circulation.

In the current study, although we found that lactating women with higher DII had more bone loss, there was no statistically significant liner trend in the maternal bone loss at 6 months postpartum. The following possible reasons for this result should be taken into account: firstly, subject is one of important matters. Postmenopausal women tended to be the focus of researchers. Participants in our study were lactating women, obviously different from those whose average age was over 65 in previous studies published in some aspects including the special physical status, life styles, and diet habits of lactating women, which might help to account for the discrepancies between ours and results above mentioned. Secondly, compared with postmenopausal women, lactating women usually have more complicated mechanisms regulating hormones related to moderating bone metabolism. Remarkable fluctuation in hormones of lactating women could weaken or amplify the inflammatory effects of diet, which leads to the divergent results. Lastly, due to the distinct genetic background, the differences in susceptibility should not be ignored.

### Limitations

We should acknowledge that the study had several limitations. Firstly, the participants were recruited in the selected maternity hospital, which limited the extrapolation of our conclusion. Secondly, although the FFQ used in the study was modified on the basis of the questionnaire applied in the Chinese Nutrition and Health surveillance, it was not revalidated, which reduced the credibility and reliability of the modified FFQ questionnaire. What’s more, QUS was used for measuring BD rather than the dual energy X-ray absorptiometry (DXA). Although DXA is always recommended as the golden diagnosis criteria of osteoporosis, it has been reported that the direct comparison of DXA with QUS of the calcaneus showed a positive correlation between both methods [52, 53] and QUS can be used to well predict the risk of fracture [53–55]. On the other hand, QUS was free of ionizing radiation, therefore is more accessible to some special population such as pregnant women, children and lactating mothers. In addition, dietary regulation of inflammatory status is a long-term and subtle process. In our study, the duration of follow up may be too short to exert detectable effect on BD. Finally, the data on dietary intake and physical activity level were recalled by participants, which inevitably caused some recall bias.

## Conclusions

Chinese women in the postpartum period with higher DII scores have more bone mass loss; however significant differences and trends are attenuated and/or disappear depending on covariates and confounders taken into account in statistical analysis. The further study should be conducted to enlarge the sample size to see whether the significant association between DII and maternal BD exists among Chinese lactating women and to collect blood samples of participants for testing the inflammatory markers so as to further testify the validation of DII in reflecting the inflammatory potential of diets in Chinese lactating women.

## Data Availability

The datasets used during the current study are available from the corresponding author on reasonable request.

## Abbreviations

BD: Bone Density
BMI: Body Mass Index
DII: Dietary Inflammatory Index (DII)
DP: Dietary pattern
DXA: Dual energy X-ray absorptiometry
FFQ: Food Frequency Questionnaires
MET: Metabolic equivalent
QUS: Quantitative ultrasonometry
